# Impact of Decipher on use of post‐operative radiotherapy: Individual patient analysis of two prospective registries

**DOI:** 10.1002/bco2.70

**Published:** 2021-01-24

**Authors:** Mohammed Shahait, Vinnie Y. T. Liu, Neha Vapiwala, Priti Lal, Jessica Kim, Eduard J. Trabulsi, Huei‐Chung Huang, Elai Davicioni, Darby J. S. Thompson, Daniel Spratt, Robert B. Den, David I. Lee

**Affiliations:** ^1^ Department of Surgery King Hussein Cancer Center Amman Jordan; ^2^ Decipher Biosciences San Diego CA USA; ^3^ Department of Radiation Oncology Perelman School of Medicine University of Pennsylvania Philadelphia PA USA; ^4^ Department of Pathology Perelman School of Medicine University of Pennsylvania Philadelphia PA USA; ^5^ Department of Surgery University of Pennsylvania Philadelphia PA USA; ^6^ Thomas Jefferson University Hospital Philadelphia PA USA; ^7^ EMMES Canada Vancouver BC Canada; ^8^ University of Michigan Ann Arbor MI USA; ^9^ Penn Urology Penn Presbyterian Medical Center Philadelphia PA USA

**Keywords:** biomarkers, prostate cancer, radiation

## Abstract

**Objective:**

To assess the association between Genomic Classifier (GC)‐risk group and post‐radical prostatectomy treatment in clinical practice.

**Methods:**

Two prospective observational cohorts of men with prostate cancer (PCa) who underwent RP in two referral centers and had GC testing post‐prostatectomy between 2013 and 2018 were included. The primary endpoint of the study was to assess the association between GC‐risk group and time to secondary therapy. Univariable (UVA) and multivariable (MVA) Cox proportional hazards models were constructed to assess the association between GC‐risk group and time to receipt of secondary therapy after RP, where secondary therapy is defined as receiving either RT or ADT after RP.

**Results:**

A total of 398 patients are included in the analysis. Patients with high‐GC risk were more likely to receive any secondary therapy (OR: 6.84) compared to patients with low/intermediate‐GC risk. The proportion of high‐GC risk patients receiving RT at 2 years post‐RP was 31.5%, compared to only 6.3% among the low/intermediate‐GC risk patients.

**Conclusion:**

This study demonstrates that physicians in routine practice used GC to identify high risk patients who might benefit the most from secondary treatment. As such, GC score was independent predictor of receipt of secondary treatment.

## 1 BACKGROUND

In concert with the increased proportion of patients diagnosed with high‐risk PCa after the USPSTF recommendation against prostate cancer screening in 2012, the utilization of radical prostatectomy (RP) for high‐risk and locally advanced prostate cancer has increased.^1^, ^2^ The risk of biochemical recurrence afterward ranges between 40% and 70%, which is associated with an increased risk of future metastasis.^3^ Post‐operative radiation plays a pivotal role in improving progression‐free survival, as well as overall survival in patients with locally advanced disease.^4^, ^5^, ^6^, ^7^, ^8^

Intriguingly, high‐risk patients with a projected life expectancy of more than 10 years received adjuvant radiation therapy (aRT) only 30% of the time.^9^ The arguments against aRT administration are: the high number needed to treat, increased treatment‐related toxicity, and decreased quality‐adjusted life expectancy.^10^ Therefore, a more sagacious approach of surveillance and administering early salvage radiation (sRT), when prostate specific antigen (PSA) is detectable but ideally less than 0.5 is more embraced among the urological community.^11^ The evidence to support the use of early sRT is based on three recently presented randomized trials, RADICALS (ISRCTN40814031), GETUG‐AFU 17 (NCT00667069) and RAVES (NCT00860652), which showed that early sRT is not inferior to aRT.^12^, ^13^, ^14^

The use of Genomic Classifier (GC) data in the post‐radical prostatectomy have been shown to predict the risk of metastasis and prostate cancer‐specific mortality, as well as improve the accuracy of risk stratification of high‐risk prostate cancer.^15^ Hence, it identifies who might benefit from aRT, which might impact patient as well as physician decision‐making regarding additional treatment.^16^

In this study, we aim to assess the association between GC risk group and post‐radical prostatectomy treatment in real world practice via utilization of two contemporary prospective cohorts of men treated with RP.

## 2 METHODS

### 2.1 Study cohorts

This study adheres to the REMARK criteria for the evaluation of prognostic biomarkers.^17^

This study included two prospective observational cohorts of men diagnosed with prostate cancer (PCa) that were treated with RP and had available post‐RP GC results. GC testing was offered to patients with adverse pathological features on the final pathology (eg, positive surgical margin, presence of extraprostatic extension, seminal vesicle invasion, or PSA persistence).^18^ The first cohort is the University of Pennsylvania (UPenn) cohort, which is part of a prospectively collected Institutional Review Board (IRB) approved comprehensive database for Robot‐Assisted Radical Prostatectomy (RARP) maintained by the corresponding author. The second is the Thomas Jefferson University (TJU) cohort, which is an IRB approved prospective cohort that implemented GC‐based treatment recommendations.^19^

The UPenn cohort consisted of 352 patients who underwent RARP between 2013 and 2018, while the TJU cohort consisted of 135 patients treated by RP between 2014 and 2016. In the UPenn cohort, the decision and the timing to administer adjuvant/early salvage radiation therapy and androgen deprivation therapy (ADT) were based on patient co‐morbidities and life expectancy, patients’ treatment expectations, PSA kinetics, and consensus of the prostate cancer multidisciplinary team. In the TJU cohort, the decision and timing to administer aRT, with or without ADT, was based on tumor board recommendations pertaining to the genomic risk score.^17^ Patients were excluded from analysis if they developed biochemical recurrence before the GC test, had lymph node invasion, PSA persistence, missing follow‐up, or pathologic information, or if they received any neoadjuvant therapy or non‐concurrent adjuvant ADT, defined as receiving ADT more than 6 months before RT. (Figure S1).

### 2.2 GC and CAPRA‐S

Decipher test results were collected prospectively in the registry, but in order to compare GC continuous score across versions of the test, GC scores were retrieved directly from the Decipher Genomics Resource Information Database (GRID). GC scores were calculated based on the predefined 22‐marker Decipher classifier.^20^ The GC score is a continuous score between 0 and 1, with the lowest scores indicating a lower risk of metastasis. GC score categorical low‐, intermediate‐, or high‐risk were based on pre‐specified cut‐points of <0.45, 0.45‐0.6, or >0.6, respectively.^15^ The Cancer of the Prostate Risk Assessment Post‐Surgical (CAPRA‐S) scores were calculated using pre‐operative PSA, pathological Gleason score, surgical margin status, extraprostatic extension, seminal vesicle invasion, and lymph node invasion.^21^ The CAPRA‐S score is an ordinal score between 0 and 12, with the lowest scores indicating a lower risk of PCa recurrence post‐RP. Patients were categorized as low‐, intermediate‐, or high‐risk based on CAPRA‐S scores of ≤2, 3‐5, or ≥6, respectively.

### 2.3 Endpoints

The primary endpoint of the study was a priori chosen as time to receipt of secondary therapy after RP, where the objective was to assess the association between GC‐risk group and time to secondary therapy. Secondary therapy is defined as receiving either RT or ADT after RP and time to secondary therapy is calculated from time of RP until event (the first occurrence of RT or ADT) or last follow‐up. A secondary endpoint was time to biochemical failure (BF) or receipt of salvage ADT (sADT). Biochemical failure is defined as PSA ≥ 0.2 ng/mL post‐RP secondary therapy. Salvage ADT is defined as receiving ADT after biochemical recurrence (BCR) or (at least 6 months) after RT, whereas ADT administered within 6 months of RT was considered concurrent therapy. Time to biochemical failure or receipt of sADT is calculated from time of RP until event (the first occurrence of BF or sADT) or last follow‐up. aRT was defined by the initiation of radiation prior to BCR, while sRT was defined by the initiation of radiation after BCR.

### 2.4 Statistical analysis

Descriptive statistics of the two cohorts combined are reported by medians and interquartile‐ranges (IQR) or frequencies and proportions, as appropriate. Distribution of radiation therapy usage across CAPRA‐S risk groups were compared using Fisher's exact test within GC risk groups.^22^ Kaplan‐Meier cumulative incidence curves of the receipt of secondary therapy risk were constructed and compared using the log‐rank test.^23^ Univariable (UVA) and multivariable (MVA) Cox proportional hazards models, stratified by institution, were used to evaluate the prognostic value of GC scores and individual clinicopathologic risk factors in predicting the risk of receiving secondary therapy after RP.^24^ Age at RP and (log2 transformed) pre‐operative PSA were treated as continuous variables while pathological Gleason grade group (4‐5 vs 1‐3), extraprostatic extension, seminal vesicle invasion, and surgical margin status were treated as categorical variables. Similarly, UVA and MVA Cox proportional hazards models were used to evaluate the prognostic value of GC scores, RT, as a time‐dependent covariate, and CAPRA‐S scores in predicting the risk of experiencing biochemical failure or receiving salvage ADT.^25^ RT and CAPRA‐S (high vs low/intermediate) were treated as categorical variables. A comparison of aRT and sRT was not performed due to the small sample size of patients treated with sRT in this cohort. Patients with missing GC scores were dropped from the analyses if the information was required. Firth's penalization method was performed to account for the small number of events.^26^

Missing time‐to‐RT or ADT (n = 11 and 9, respectively) values were assumed to be missing at random and imputed with the respective median times. In a sensitivity analysis, the missing values were imputed with a random sample of the respective time‐to‐RT or ‐ADT variables. In 1,000 runs, results remained consistent with 100% of the hazard ratios in the same direction and 100% of the p‐values remaining significant. Hazard ratios of GC were reported per 0.1‐unit increase in score or as categorical (high vs. low/intermediate). All statistical analyses were performed in R version 3.6.1 (R foundation, Vienna, Austria) and all statistical tests were two‐sided, using a 5% significance level.

## 3 RESULTS

### 3.1 Patient characteristics

GC was ordered for 352 patients in the UPenn cohort and 135 patients in the TJU cohort considering therapy options after RP. Fifty‐four patients with detectable PSA, 12 patients with lymph node invasion or lymph nodes that could not be evaluated, three patients with negative surgical margins and pT2 stage, and one patient with BCR prior to ordering GC testing were excluded from study. In addition, four patients with incomplete pathologic information, six patients with missing follow‐up information, seven patients whose GC test or genomic samples could not be retrieved from Decipher GRID, and two patients who received adjuvant ADT were excluded, resulting in a total of 398 patients for analysis (Figure S1).

**FIGURE 1:**
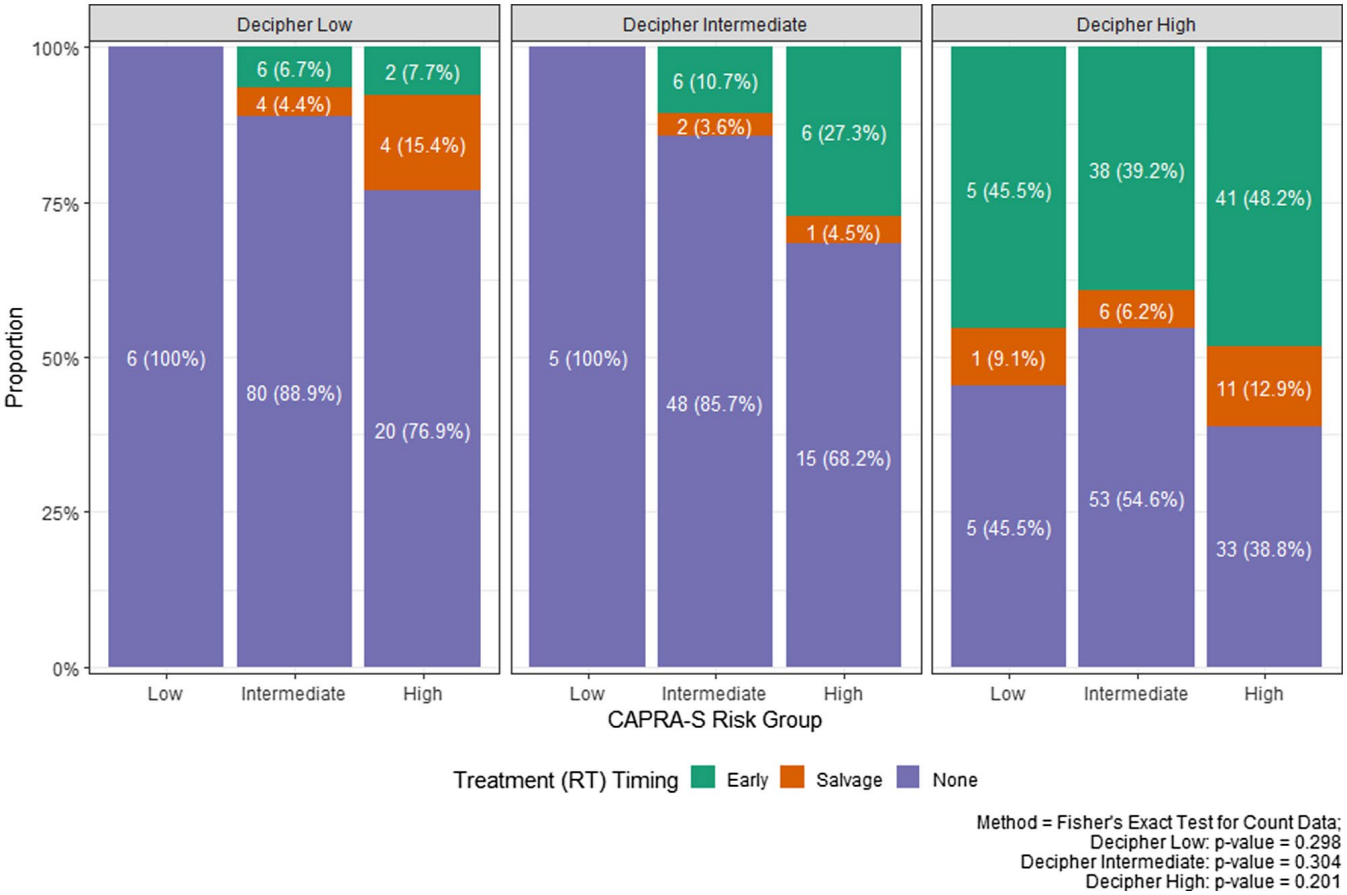
Radiation therapy administration, demonstrating distribution of earlier radiotherapy treatment and reclassification of Decipher risk and CAPRA‐S risk groups, compared using Fisher's exact test for count data. CAPRA‐S, Cancer of the Prostate Risk Assessment Post‐Surgical; RT radiation therapy

Table 1 summarizes the demographic and clinicopathological characteristics of the final cohort. The median patient age at RP was 64 (IQR: 58‐68) with a median follow‐up of 2 years (see Table S1 for the summary of events and follow‐up times). The median PSA at RP was 5.8 (IQR: 4.53‐8.48) ng/ml. On the final pathology, 16.6% had Gleason grade group 4‐5, 61.6% had an extraprostatic extension, 17.1% had seminal vesicle invasion, and 83.9% had a positive surgical margin. The median GC score of the cohort was 0.593 (IQR: 0.022‐0.977), which classified into 30.7%, 20.9%, and 48.5% of patients as low‐, intermediate‐, and high‐GC risk, respectively. Only 5.5% of men were categorized as low‐risk using the CAPRA‐S prognostic model. Similarly, 50% (11/22) of patients with low‐risk CAPRA‐S were found to have high‐GC scores while 19.5% (26/133) of patients with high‐risk CAPRA‐S were found to have low‐GC scores (Figure 1).

**TABLE 1:** Demographic and clinical characteristics of the combined UPenn and TJU cohort

Variables	GC low/intermediate	GC high	Full cohort
No. patients (%)	205 (25.8)	193 (24.2)	398 (50.0)
Age at RP			
Median (Q1, Q3)	63 (58, 67)	64 (58.5, 68.1)	63.6 (58, 68)
Pre‐op PSA			
Median (Q1, Q3)	5.7 (4.5, 8.1)	5.85 (4.7, 9.2)	5.8 (4.53, 8.48)
Path Gleason grade group			
1	6 (2.9)	1 (0.5)	7 (1.8)
2	129 (62.9)	77 (39.9)	206 (51.8)
3	55 (26.8)	64 (33.2)	119 (29.9)
4	13 (6.3)	29 (15.0)	42 (10.6)
5	2 (1.0)	22 (11.4)	24 (6.0)
Extraprostatic extension			
Yes	113 (55.1)	132 (68.4)	245 (61.6)
Seminal vesicle invasion			
Yes	22 (10.7)	46 (23.8)	68 (17.1)
Positive surgical margins			
Yes	180 (87.8)	154 (79.8)	334 (83.9)
CAPRA‐S			
Median (Q1, Q3)	4 (3, 5)	5 (4, 7)	5 (3, 6)
Categorical CAPRA‐S			
Low	11 (5.4)	11 (5.7)	22 (5.5)
Intermediate	146 (71.2)	97 (50.3)	243 (61.1)
High	48 (23.4)	85 (44.0)	133 (33.4)
GC			
Median (Q1, Q3)	0.419 (0.328, 0.525; NA = 6)	0.725 (0.661, 0.819; NA = 8)	0.593 (0.413, 0.719; NA = 14)
Categorical GC			
Low	122 (59.5)		122 (30.7)
Intermediate	83 (40.5)		83 (20.9)
High		193 (100.0)	193 (48.5)
Treated with RT			
Adjuvant (before BCR)	20 (9.8)	84 (43.5)	104 (26.1)
Salvage (after BCR)	11 (5.4)	18 (9.3)	29 (7.3)
None	174 (84.9)	91 (47.2)	265 (66.6)
ADT only			
Yes		4 (2.1)	4 (1.0)
Follow‐up time for censored patients (yrs)			
Median (Q1, Q3)	2.01 (1.39, 2.91)	1.83 (1.02, 2.38)	2 (1.17, 2.69)

Abbreviations: ADT, androgen deprivation therapy; CAPRA‐S, cancer of the prostate risk assessment post‐surgical; PSA, prostate specific antigen; RP, radical prostatectomy; RT, radiation therapy.

### 3.2 GC‐risk and receipt of secondary therapy

A total of 33.4% (133/398)of the full cohort received RT at a median of 12.6 months after RP (IQR: 7.5‐20). Correspondingly, 26.1% (104/398) of the full cohort received aRT (prior to BCR) and 10.6% (42/398) received concurrent androgen deprivation therapy with radiation therapy. Overall, utilization of therapy was higher among high‐GC risk patients, reflected in the likelihood of a patient with high‐GC risk to receive any secondary therapy being 6.84 times that of a patient with low/intermediate‐GC risk (Table S2). Specifically, the proportion of high‐GC risk patients receiving radiation therapy at 2 years post‐RP was higher, with 31.5% of such patients receiving RT compared to only 6.3% among the low/intermediate‐GC risk patients. At any given time over 5 years of post‐RP follow‐up, the proportion of high‐GC risk patients receiving RT was consistently higher than the proportion among the low/intermediate‐GC patients, indicating an overall higher utilization of radiation therapy (Figure S2, panels B [low/intermediate‐GC] and C [high‐GC]).

**FIGURE 2:**
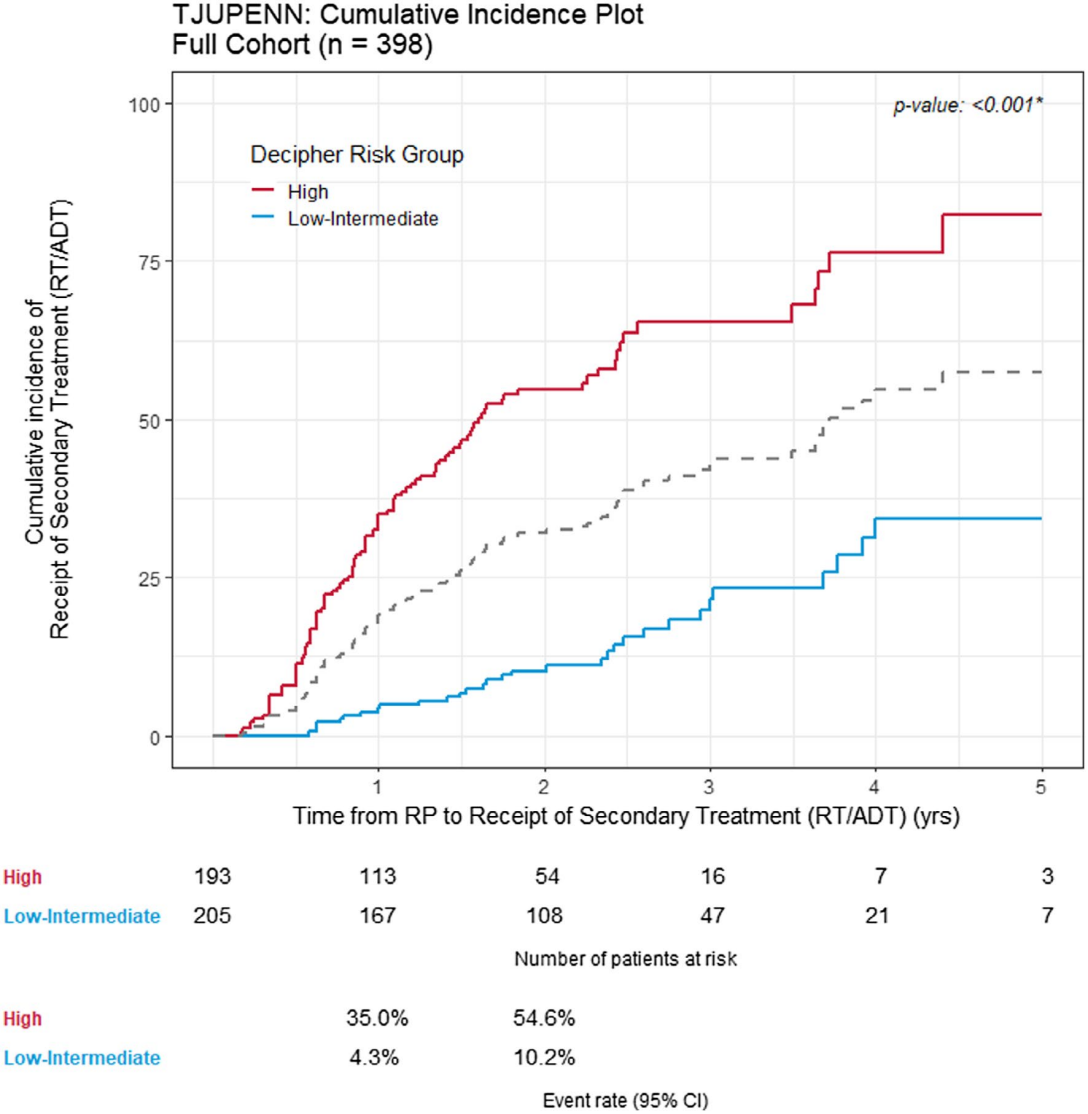
Cumulative incidence of the receipt of secondary therapy stratified by GC risk, compared using the log‐rank test. ADT, androgen deprivation therapy, RP, radical prostatectomy, RT, radiation therapy

**FIGURE 3:**
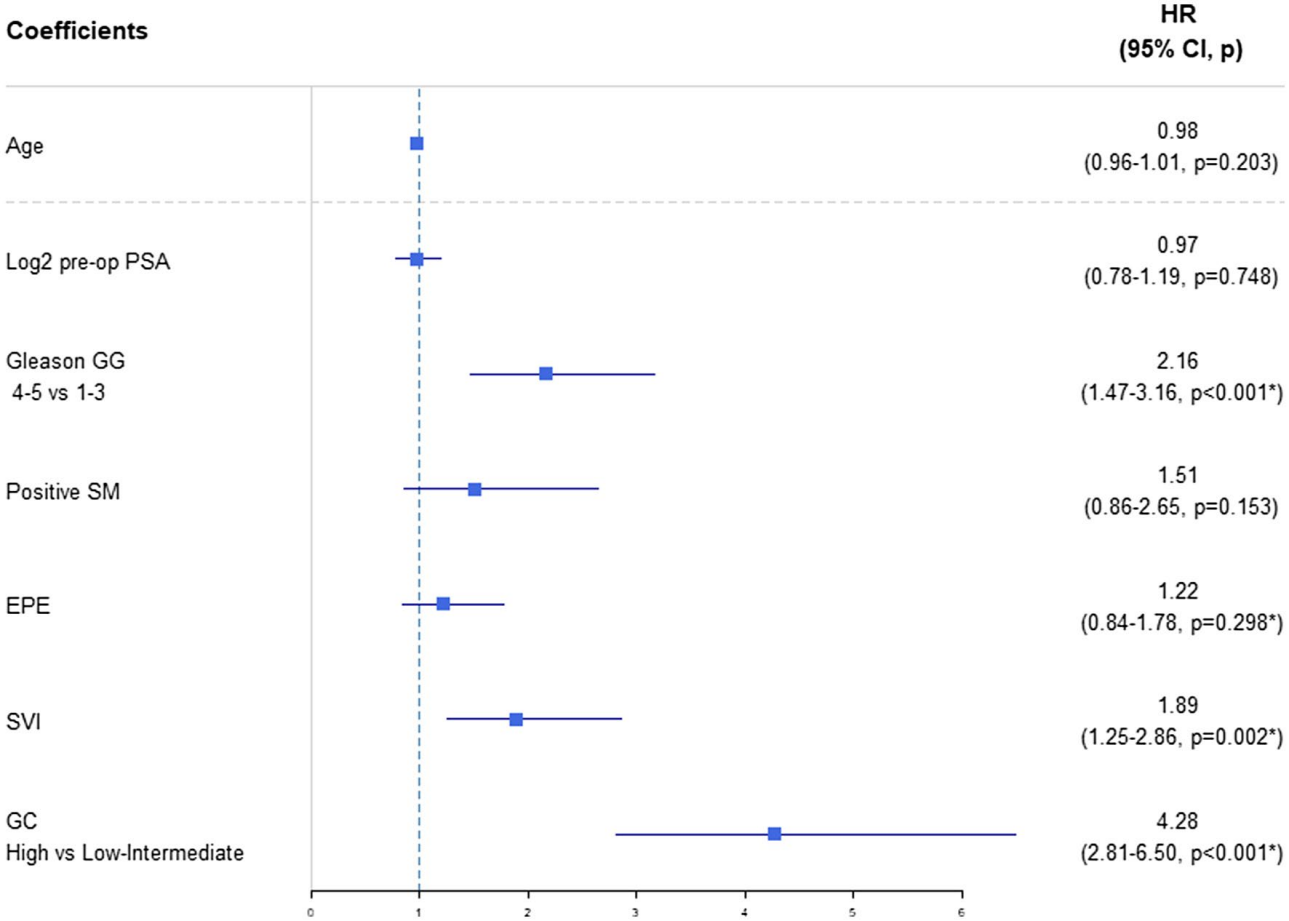
MVA Cox proportional hazards model predicting receipt of secondary therapy (RT/ADT). EPE, extraprostatic extension; GC, genomic classifier; GG, grade group; PSA, prostate specific antigen; SM, Surgical margins; SVI, seminal vesicle invasion

Overall, secondary therapy was administered at different rates when stratified by GC risk (Figure 2, *P* < .001), where high‐GC risk patients had a 2‐year cumulative incidence of receiving secondary therapy of 54.6% compared to 10.2% among the low/intermediate‐GC risk patients. On the multivariable analysis, GC score was an independent predictor of receipt of secondary therapy. Compared to patients with low/intermediate‐GC Risk, patients with high‐GC risk had a significantly higher chance of receiving secondary therapy (Figure 3, HR: 4.28 [2.81−6.50], *P* < .001). Similar results were seen with the continuous GC score (Table S3).

### 3.3 Timing of RT for different GC‐risk groups and biochemical failure or receipt of salvage ADT

**FIGURE 4:**
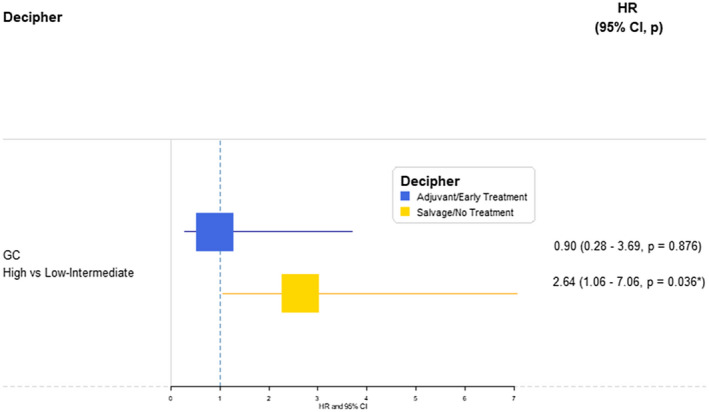
Firth's corrected MVA Cox proportional hazards model predicting biochemical failure or receipt of salvage ADT, adjusting for time‐dependent receipt of RT and CAPRA‐S, among the patients treated with adjuvant/early RT and patients treated with salvage RT or no RT at all. CAPRA‐S, Cancer of the Prostate Risk Assessment Post‐Surgical; GC, genomic classifier, RT, radiation therapy

Overall, patients developed BF or received sADT at different rates when stratified by GC risk (Figure S3, *P* < .001), where high‐GC risk patients had a 2‐year cumulative incidence of 12.5% compared to 2.2% among low/intermediate‐GC risk patients. However, patients with high‐GC risk who received aRT had no greater risk of developing BF or receiving sADT compared to patients with low/intermediate‐GC risk after adjusting for RT as a time‐dependent covariate and CAPRA‐S. Conversely, patients with high‐GC risk who did not receive early RT before BCR (ie, received salvage RT or no RT at all) had significantly greater risk of progressing into BF or receiving sADT compared to patients with low/intermediate‐GC risk (Figure 4, HR: 2.64 [1.06‐7.06], *P* = .036). In the subset of men who did not receive RT on study follow‐up, patients with high‐GC risk had a much higher risk of receiving sADT (HR: 16.47 [1.65‐2215.14], *P* = 0.014) compared to low/intermediate‐GC risk.

## 4 DISCUSSION

In this study, we found that patients with high‐GC risk were 6.84 times more likely to receive any secondary therapy compared to patients with low/intermediate‐GC risk and that GC score was an independent predictor of receiving secondary therapy. This shows that more physicians use GC score not only for risk stratification and prognostication but to guide post‐RP management in the real world. Recently, Marascio et al. examined the clinical utility of GC testing on post‐radical prostatectomy management and found that GC testing changed the treatment plan for 39% of the patients, which translated to a tenfold reduction in the risk of biochemical recurrence in patients with high‐GC risk who received aRT.^18^ Correspondingly, 76.7% of the patients in this cohort who received post‐op RT were classified as high‐GC risk, which translated into a decrease in the risk of developing BF or receiving sADT among high‐GC risk patients who received aRT.

The clinical utility of the GC test has been observed in the PRO‐IMPACT prospective trial and in the Medicare Certification and Training registry cohort.^27^ However, its applicability to the daily practice has been debated. In this report, we confirm the versatility of GC testing in the risk stratification of patients and in guiding post‐RP management in two different prospective cohorts from two different institutions.

Interestingly, we found that aRT mitigated the effect of GC on the risk of developing BF or receiving sADT. In this study we sought to use BF as a surrogate endpoint for analysis, as it is highly associated with future risk of metastasis.^28^ The ARTISTIC meta‐analysis of three randomized trials, RADICALS (ISRCTN40814031), GETUG‐AFU 17 (NCT00667069), and RAVES (NCT00860652), showed that early sRT is not inferior to aRT.^29^ It is noteworthy that these trials failed to account for the molecular heterogeneity of prostate cancer. Additionally, the number of patients with multiple adverse pathological features was low, which affected the applicability of the results from these trials on a very high‐risk population with multiple adverse pathological features and high‐GC risk. Indeed, these individuals are most likely to benefit from aRT, rather than sRT, as previously demonstrated by Dalela et al.^30^

Our study has several limitations that warrant discussion. First, the short median follow‐up of 2 years necessitated analysis of post‐operative treatment failure, a surrogate endpoint, rather than a proven surrogate endpoint for survival, such as metastasis. Moreover, the number of patients who received sRT was small and the fact that this was an observational registry (ie, treatment was not randomly assigned, but based on baseline clinical and genomic risks) did not allow us to do a formal comparison with aRT. Finally, GC testing at UPenn was offered to all patients with pT3 disease, and pT2 with positive surgical margins; however, some patients declined the test due to lack of insurance coverage.

The current guidelines have emphasized the importance of aRT in improving progression‐free survival as well as overall survival in patients with locally advanced disease.^7^, ^8^ Nevertheless, RT is associated with increased treatment‐related toxicity and decreased quality‐adjusted life expectancy. Therefore, only about 30% of high‐risk PCa patients receive aRT after RP, which leads to undertreatment for a substantial number of high‐risk patients and compromises their outcomes.^30^ This study demonstrates that physicians in the daily practice used GC to identify high risk patients who might benefit the most from aRT, notwithstanding the nuances in the interpretation of GC score impact and their post‐prostatectomy management approach. Optimal post‐operative secondary treatment decision making should integrate clinical and genomic risk factors. Longer follow up from these registries and other ongoing studies will help elucidate the impact of these decisions on clinical outcomes.

## Supporting information

Fig S1

Fig S2

Fig S3

Table S1

Table S2

Table S3
