# Supplementary material for: Impact of Decipher on use of post‐operative radiotherapy: Individual patient analysis of two prospective registries
Source: BJUI Compass. 2021 Jan 24;2(4):267–74. doi: 10.1002/bco2.70 (PMC8988525; doi:10.1002/bco2.70)
Supplement: Supplementary file 1 — Fig S1 [file BCO2-2-267-s004.docx]

Supp Figure S1:


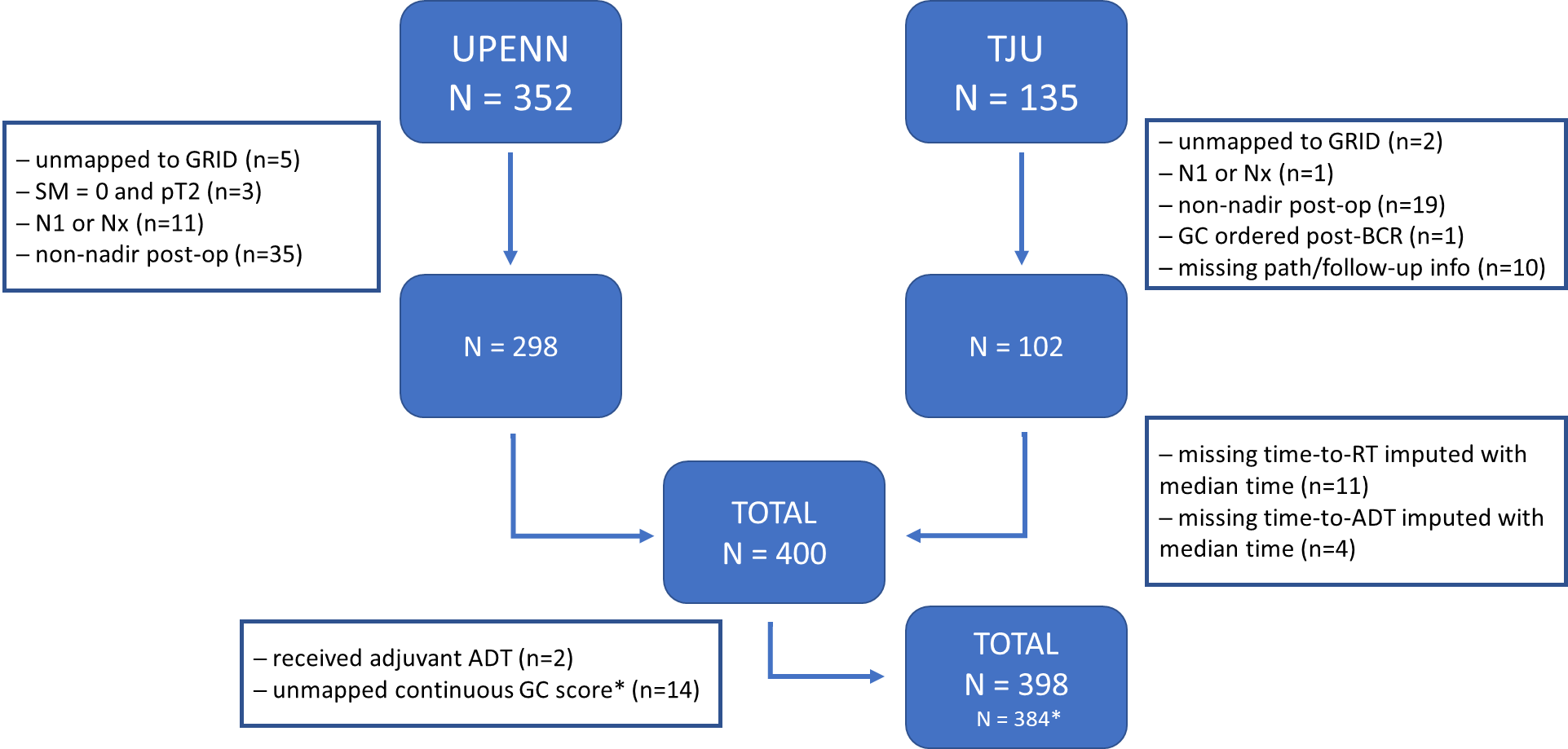


Figure S1: Consort diagram showing the patient selection criteria for the UPenn and TJU cohorts. GRID Genomics Resource Information Database, SM surgical margins, BCR biochemical recurrence, ADT androgen deprivation therapy.
