# Supplementary material for: Impact of Decipher on use of post‐operative radiotherapy: Individual patient analysis of two prospective registries
Source: BJUI Compass. 2021 Jan 24;2(4):267–74. doi: 10.1002/bco2.70 (PMC8988525; doi:10.1002/bco2.70)
Supplement: Supplementary file 2 — Fig S2 [file BCO2-2-267-s005.docx]

Supp Figure S2:


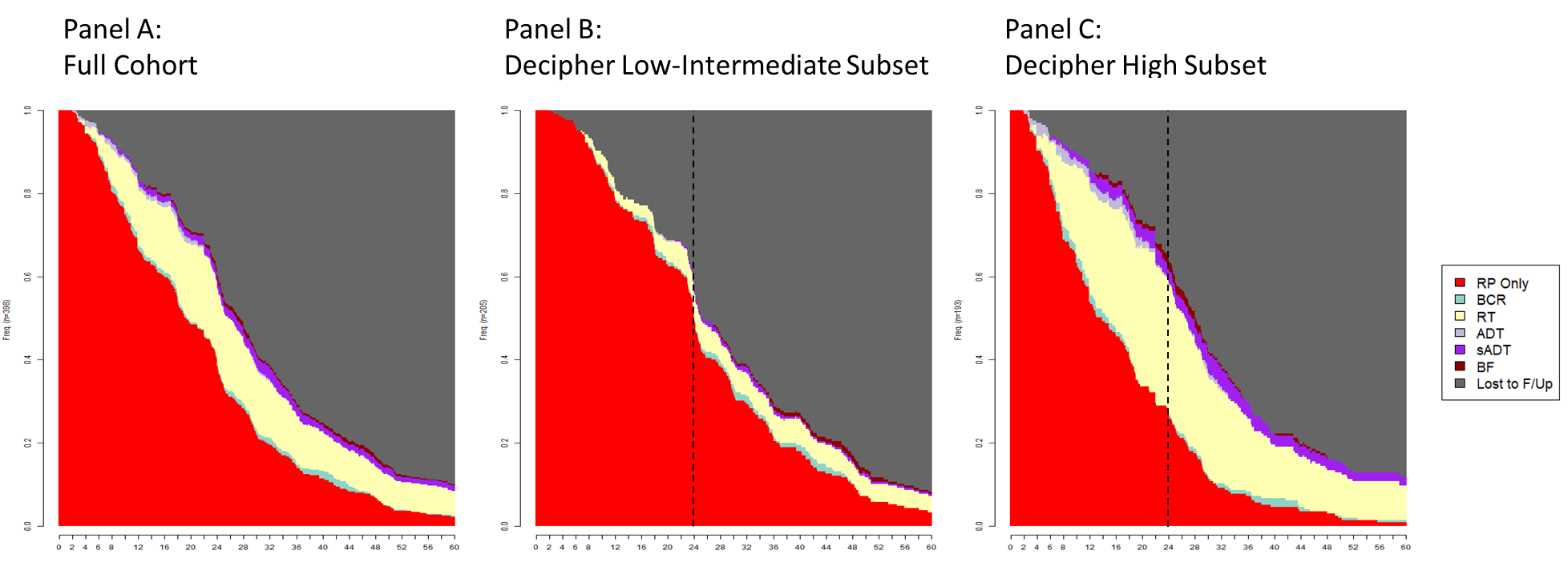


Figure S2: Cumulative treatment trend illustrating the proportion of patients falling into each treatment state at each time point (from time of RP up to 5 years of follow-up) in **a)** the full cohort, **b)** the subset of low/intermediate-GC risk patients, and **c)** the subset of high-GC risk patients. RP radical prostatectomy, BCR biochemical recurrence, RT radiation therapy, ADT androgen deprivation therapy, sADT salvage androgen deprivation therapy, BF biochemical failure, F/Up follow-up.
