# Supplementary material for: Impact of Decipher on use of post‐operative radiotherapy: Individual patient analysis of two prospective registries
Source: BJUI Compass. 2021 Jan 24;2(4):267–74. doi: 10.1002/bco2.70 (PMC8988525; doi:10.1002/bco2.70)
Supplement: Supplementary file 3 — Fig S3 [file BCO2-2-267-s002.docx]

Supp Figure S3:


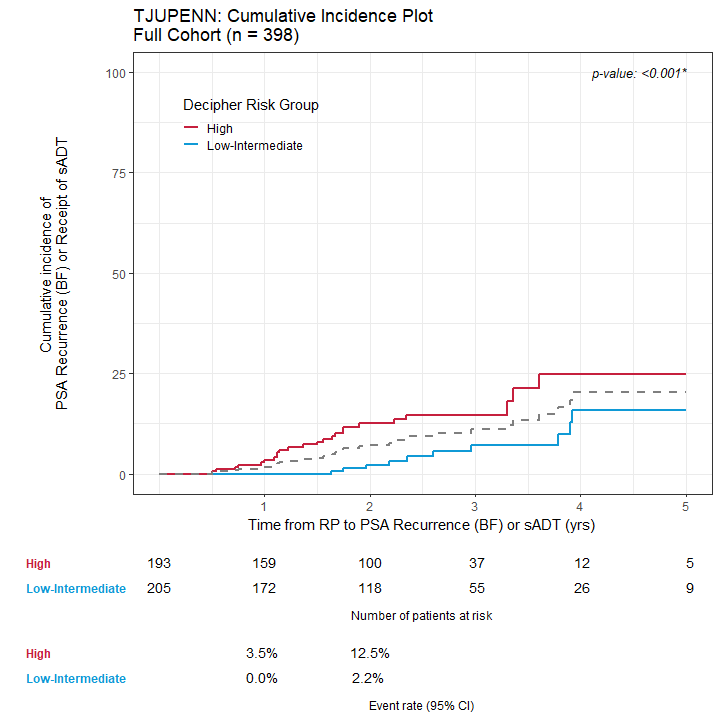


Figure S3: Cumulative incidence of biochemical failure or receipt of salvage ADT stratified by GC risk, compared using the log-rank test. RP radical prostatectomy, PSA prostate specific antigen, BF biochemical failure, sADT salvage androgen deprivation therapy
