# Supplementary material for: Impact of Decipher on use of post‐operative radiotherapy: Individual patient analysis of two prospective registries
Source: BJUI Compass. 2021 Jan 24;2(4):267–74. doi: 10.1002/bco2.70 (PMC8988525; doi:10.1002/bco2.70)
Supplement: Supplementary file 4 — Table S1 [file BCO2-2-267-s001.docx]

| **Variables** | **GC Low/Intermediate** | **GC High** | **Full Cohort** |
| --- | --- | --- | --- |
| **No. Patients (%)** | 205 (25.8) | 193 (24.2) | 398 (50.0) |
| **Biochemical Recurrence Post-Op** |  |  |  |
| Yes | 17 (8.3) | 24 (12.4) | 41 (10.3) |
| **Time to Biochemical Recurrence Post-Op** |  |  |  |
| Median (Q1, Q3) | 30 (17.2, 40.5) | 12 (7.43, 28) | 24.1 (10, 37) |
| **Follow-up Time for censored patients**  **(to Biochemical Recurrence Post-OP)** |  |  |  |
| Median (Q1, Q3) | 24.4 (17, 35.4) | 24.9 (17, 32.6) | 24.6 (17, 34) |
| **Biochemical Failure** |  |  |  |
| Yes | 3 (1.5) | 10 (5.2) | 13 (3.3) |
| **Time to Biochemical Failure** |  |  |  |
| Median (Q1, Q3) | 35.4 (31.2, 40.5) | 25.4 (20.6, 38.5) | 28 (22.5, 39.6) |
| **Follow-up Time for censored patients**  **(to Biochemical Failure)** |  |  |  |
| Median (Q1, Q3) | 24.8 (17.5, 37.1) | 24.9 (17, 33.3) | 24.8 (17, 35.4) |
| **Receipt of Secondary Therapy (RT +/- ADT)** |  |  |  |
| Yes | 31 (15.1) | 106 (54.9) | 137 (34.4) |
| **Time to Secondary Therapy** |  |  |  |
| Median (Q1, Q3) | 20.9 (12, 32.1) | 10.8 (6.83, 17.3) | 12 (7.5, 19.6) |
| **Follow-up Time for censored patients**  **(to Secondary Therapy)** |  |  |  |
| Median (Q1, Q3) | 24.3 (17, 35.4) | 22.3 (12.5, 29) | 24 (14.9, 33.1) |
| **Biochemical Failure or Receipt of sADT** |  |  |  |
| Yes | 10 (4.9) | 25 (13.0) | 35 (8.8) |
| **Time to BF or Receipt of sADT** |  |  |  |
| Median (Q1, Q3) | 29.7 (24.1, 43) | 18 (13, 22.8) | 21 (13.5, 29.7) |
| **Follow-up Time for censored patients**  **(to Biochemical Failure or Receipt of sADT)** |  |  |  |
| Median (Q1, Q3) | 24.7 (17.2, 36.4) | 25 (17.4, 33.1) | 24.9 (17.2, 35) |
