# Supplementary material for: Impact of Decipher on use of post‐operative radiotherapy: Individual patient analysis of two prospective registries
Source: BJUI Compass. 2021 Jan 24;2(4):267–74. doi: 10.1002/bco2.70 (PMC8988525; doi:10.1002/bco2.70)
Supplement: Supplementary file 5 — Table S2 [file BCO2-2-267-s006.docx]

Table S2: Number of patients receiving any secondary therapy (RT/ADT) with GC risk group and calculated odds ratio of receipt of secondary therapy for GC High vs. Low-Intermediate. OR odds ratio

**
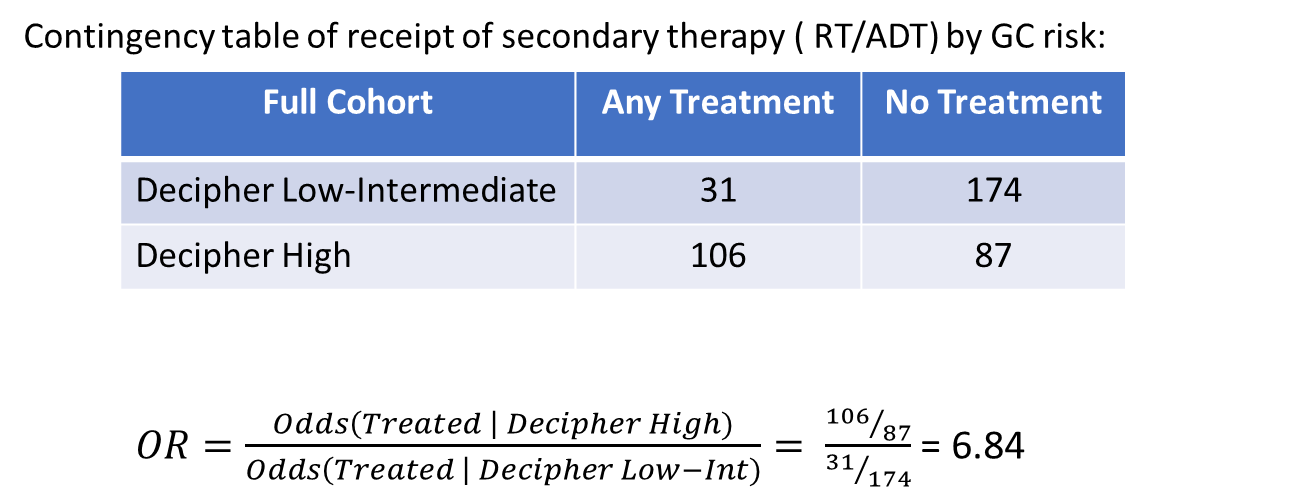
**
