# Supplementary material for: Impact of Decipher on use of post‐operative radiotherapy: Individual patient analysis of two prospective registries
Source: BJUI Compass. 2021 Jan 24;2(4):267–74. doi: 10.1002/bco2.70 (PMC8988525; doi:10.1002/bco2.70)

Table S3: Univariable (UVA) and multivariable (MVA) Cox proportional hazards model for predicting receipt of secondary therapy (RT/ADT), stratified by institution. The Hazard ratio for continuous Decipher is reported per 0.1-unit increase. PSA prostate specific antigen, GG grade group


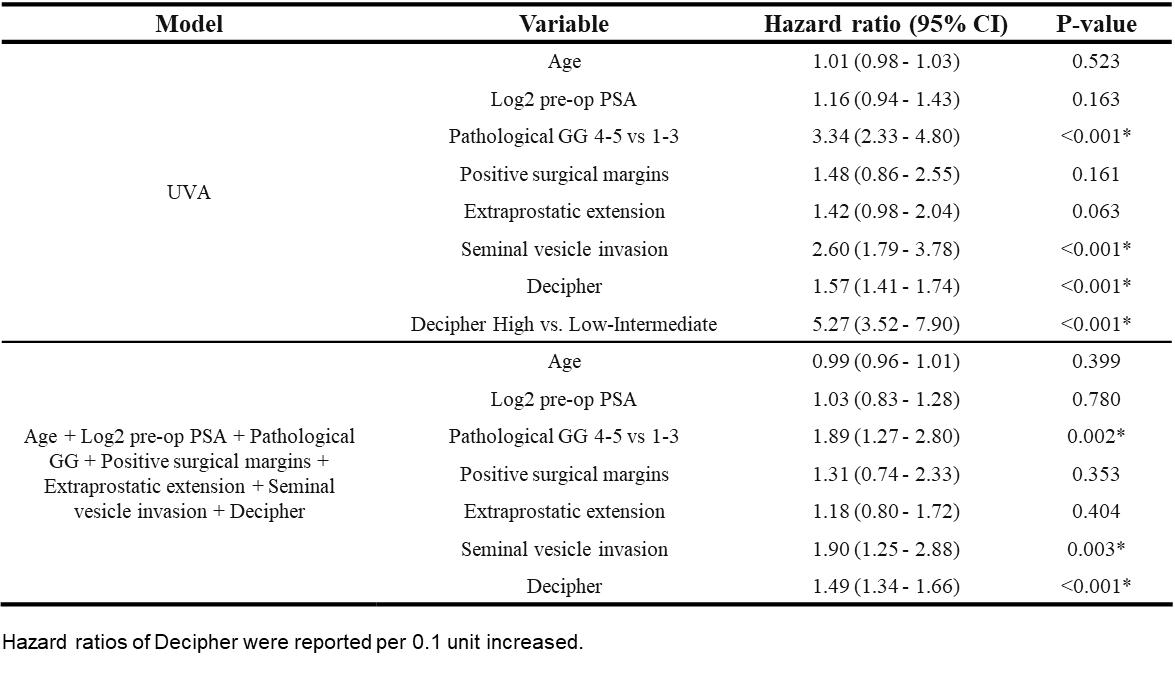

Supplement: Supplementary file 6 — Table S3 [file BCO2-2-267-s003.docx]
